# Periorbital ecchymosis and subconjunctival hemorrhage due to leech therapy for headache

**DOI:** 10.5935/0004-2749.20210057

**Published:** 2025-02-02

**Authors:** Neslihan Sevimli, Remzi Karadag, Ayse Serap Karadag

**Affiliations:** 1 Department of Ophthalmology, Sultanbeyli State Hospital, Sultanbeyli, Istanbul, Turkey; 2 Veni Vidi Eye Center, Caddebostan, Kadikoy, Istanbul, Turkey; 3 RK Eye, Aesthetics and Health Services, Kadikoy, Istanbul, Turkey; 4 Department of Dermatomology, Istanbul Medeniyet University School of Medicine, Goztepe, Kadikoy, Istanbul, Turkey

**Keywords:** Headache/therapy, Hirudo medicinalis, Leeching/ adverse effects, Orbital diseases, Hematoma, Conjunctiva, Eye hemorrhage/etiology, Cefaléia/terapia;Hirudo medicinalis;Aplicação de sanguessugas/efeitos adversos, Doenças orbitárias, Hematoma, Túnica conjuntiva, Hemorragia ocular/etiologia

## Abstract

A 62-year-old woman was admitted to our clinic with the complaints of periorbital ecchymosis and subconjunctival hemorrhage that are visible, especially on the right eye. We noted that her complaints began the day after she underwent leech therapy on the glabella area for headache. On the glabella, 2 leech bites were observed close to the right side. Examination revealed ecchymosis on the bilateral eyelids and subconjunctival hemorrhage on the inferolateral and medial limbus on the right eye. No treatment was initiated, rather control measures were recommended. The follow-up after 1 month revealed that the patient’s complaints had disappeared.

## Abstract (pt)

Uma paciente de 62 anos procurou nosso ambulatório com queixas de equimose periorbital e hemorragia subconjuntival, visíveis principalmente no olho direito. Descobrimos que suas queixas começaram no dia seguinte a um tratamento para dor de cabeça com sanguessugas na área da glabela. Na glabela, 2 mordidas de sanguessuga foram encontradas próximas ao lado direito. Durante os exames da paciente, foram detectadas equimoses nas pálpebras bilaterais e hemorragia subconjuntival no limbo ínfero lateral e medial do olho direito. Nenhum tratamento foi iniciado, sendo recomendado apenas controle. No acompanhamento, observou-se que as queixas da paciente desapareceram em cerca de um mês.

## INTRODUCTION

The leeches are intervertebrates that are used for therapeutic purposes in the conventional medicine since the past 2500 years^(1-3)^.These leeches include *Hirudomedicinalis* and the treatment is called hirudotherapy^(4-6)^.

Leech therapy is used for treating cases of plastic and reconstructive surgery, recovery of scar and flap, tumor, abscess, joints, vascular and ocular diseases, headache, ulcer, hemorrhoids, and thrombosis, among others^(5-7)^. Leeches secrete anesthetic, anticoagulant, antiplatelet, and anti-inflammatory substancesthrough their saliva^(6-8)^.

Leeches suck out the surplus blood and decrease swallowing on the tissue, thereby accelerating the recovery of scar by freshly oxidized blood^(7,8)^.This therapy may cause side-effects such as rashes, itching, infection, and bleeding on the bite area from hirudin exposure^(7)^.

Although leeches are used therapeutically in treating some ocular diseases^(4,9,10)^, they may accidentally cause ocular infection^(1-3)^.

In this case report, we aimed to present the development of periorbital ecchymosis and subconjunctival hemorrhage in patients who underwent leech therapy for headache.

## CASE REPORT

A 62-year-old women visited our clinic with the complaints of periorbital ecchymosis and rasheson both her eyes 5 days after undergoing leech therapy on the glabella area for curing her headache. The patient confirmed having developed rashes on the application site on the day of leech therapy and that she had developed ecchymosis and rashes inside the eye a day after the therapy.The patient reported no traumatic history or chronic ocular disease. She did not have any known history of allergy.

**Figure 1:**
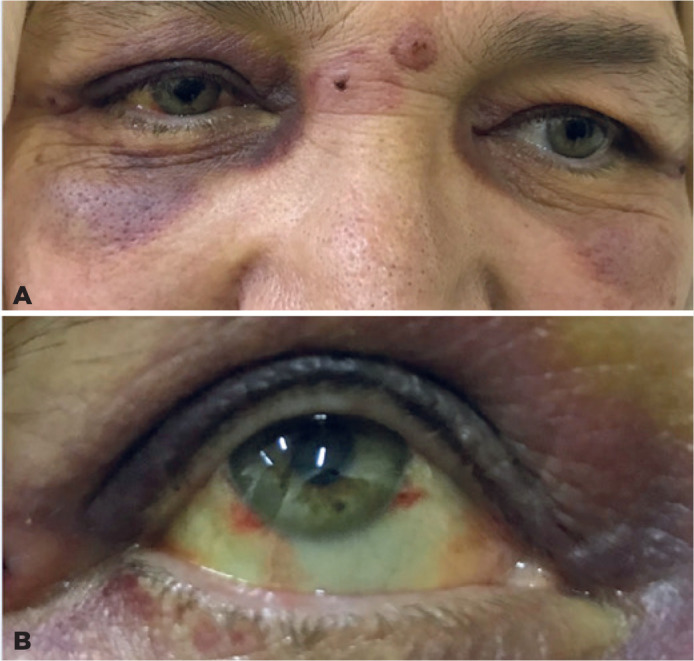
The patientpresenting with2 bite scars on the glabella and ecchymosis that areintense on the lower eyelids and at the right side (A). Subconjunctival hemorrhage on the inferomedial and inferolateral limbus on the right eye (B).

Her ocular examination revealed best-corrected visual acuity (BCVA) of 20/20 (Snellen chart) in both the eyes. Intraocular pressure (IOP) was measured as 15 mmHg on the right side and 16 mmHg on the left side. Periorbital ecchymosis was noted on the bilateral lower and upper eyelids of the right eye. Ecchymosis was found to be more intense on the lower eyelids (Figure 1A). There were 2 points visible on the glabella area where the leeches had attached, with rashes around them. Leech’s trace of bite on the right side of the glabella was closer to the right eye than to the left eye. No swelling, palpation, and pain were noted on the eyelids. The eye movements were normal. Biomicroscopic examination revealed intense subconjunctival hemorrhage on the inferomedial and inferolateral limbus of the right eye (Figure 1B). The anterior segment examination of the left eye showed normal findings. The bilateral posterior segment was normal. The vitals of the patient were stable, and she did not have any fever. The diagnosis of periorbital or orbital cellulitis was excluded based on the clinical findings. Intense ecchymosis and subconjunctival hemorrhage has been associated with leech therapy. The patient was not provided any treatments, rather control measures were recommended. In her follow-up after 1 month, no noticeable signs of damage were noted at the site of leech therapy.

## DISCUSSION

Hirudotherapy is a conventional treatment approach that has long been practiced with *H. medicinalis* leeches to cure various diseases^(1-3)^.Leeches’ saliva contains >20 biologically active substances with known properties such as anti-inflammatory, antiplatelet, anticoagulant, and antimicrobial^(6)^.The application of the saliva can heal the tissues in cases of abscess, arthritis, thrombosis, venous disorders, and headache^(6)^.

Leeches have a mouth on the front and back of their body, and they can suck blood as many as 10-times in volume relative to their size. The blood-sucking characteristics of leeches are especially helpful to evacuate the blood accumulated between the flap in plastic and reconstructive surgeries and in the treatment of edema and hematoma. In addition, this method enables easing the pain in the affected area^(5,7,9)^.

In a past study, leech therapy was successfully applied in a patient with serious periorbital hematoma depending on the penetrant injury.The practitioners found that the 6 leeches used in their study decreased the swelling on the eyelid and periorbital areas, and the eyelid was opened to examine the ocular structure^(9)^. There are other supportive publications for the successful application of leech therapy in cases of acute congestive glaucomausing the same mechanism^(1,10)^.

Infection may develop following leech therapy. *Aeromonashydrophila* lives symbiotically in the intestines of *H. medicinalis*^(5,9)^.Similar to that in our case, another study also reported orbital cellulitis in a patient who undertook leech therapy for headache^(4)^.Although the causative factor was not mentioned in this past case, we can assume it to be *A. hydrophila*^(4)^. Accordingly, the authors emphasized that this therapy must be applied under appropriate conditions by professionals only.

In the present case, no findings such as pain, fever, or general disorder suggestive of any underlying infection was noted.The patient’s blood tests showed normal complete blood count and biochemical findings.

In another study,irritant contact dermatitiswas noted presenting with the symptoms of severe rash and itching on a patient who undertook leech therapy for headache and neck pain^(5)^.Hirudinis the principal component of leech saliva responsible for the anticoagulant activity that helps prevent clotting by inhibiting thrombin. In the treatments, a great number of leeches are applied for a long time on the affected area, which may ienduce serious bleeding, resulting in anemia and requiring transfusion in places the leech attaches to the mucous^(5,6)^. In our case, periorbital ecchymosis and subconjunctival hemorrhage, which were intense especially in the injection area, may have occurred owing to the anticoagulant effect of hirudin.

The painkiller characteristics of the saliva of leeches can be attributed to the analgesic agents secreted by leeches that are reportedly fast-acting, effective, and long-standing^(6,7)^.On this basis, our patient started undertaking leech therapy for her medical concerns.

During their application for medical treatment, leeches can accidentally penetrate into the eye. Owing to the anesthetic material contained in the leeches’ saliva, the bite is painless. As a result, the patient does not realize the presence of the leeches. In patients with ocular complaints, only careful anamnesis and examination can diagnose ocular leech infestation^(6,8)^.In the present case, whether the leeches applied on the glabella area had penetrated into the eye was assessed via ophthalmoscopic examination.

Leech infestation wasobserved in a case that was primarily diagnosed with conjunctival pigmented nevus by mistake^(3)^.The authors noted that ocular leeches can be mistaken for scleral perforation, which causes uveal prolapses.

In conclusion, it is critical to practice caution with leech therapy when applied in the conventional pattern to avoid development of undesired side-effects on the eye, especially when applied close to the eye area.
